# Pergolide Mesilate May Improve Fatigue in Patients with Parkinson’s Disease

**DOI:** 10.1155/2002/473140

**Published:** 2002-11-01

**Authors:** Kazuo Abe, Mayako Takanashi, Takehiko Yanagihara, Sabro Sakoda

**Affiliations:** Department of Neurology Osaka University Graduate School of Medicine Osaka Japan

## Abstract

*Objectives:* Fatigue is a complaint frequently encountered among patients with Parkinson’s disease (PD). Considering the possible relationship between fatigue and dopaminergic dysfuncion, we investigated the effect of pergolide mesilate (a D2 and D1 dopamine receptor agonist) and bromocriptine (a D2 selective dopamine receptor) in patients with PD.

*Methods:* We evaluated 41 patients with PD and controls. We assessed the degree of fatigue by using a fatigue scale. The severity of PD was evaluated by the Hoehn and Yahr Scale and the unified Parkinson’s disease rating scale (UPDRS).

*Results:* After five weeks from prescription, patients taking pergolide mesilate showed significant improvement in the fatigue scale (from 5.1 ± 0.7 to 4.4 ± 0.55, *p* < 0.05, ) but patients taking bromocriptine did not (from 4.8 ± 0.9 to 4.7 ± 0.8).

*Conclusions:* Our study suggested the possibility of functional correlation between fatigue and D1 receptor in patients with PD.

## 1. Introduction

The typical clinical features of Parkinson's disease (PD) consist of bradykinesia, rigidity, resting tremor, and postural abnormalities [1,2]. In addition, increasing attention has been paid to other non-motor dysfunctions including cognitive dysfunction [3,4]. Although many clinicians consider fatigue to be an important feature of PD, there have been few studies of fatigue in patients with PD, and standard textbooks on PD do not list fatigue in their indexes [5].

Fatigue is a common complaint in patients with neurologic, psychiatric, and systemic diseases, and it is chronic and severe in some patients. Fatigue is defined as an overwhelming sense of tiredness, lack of energy, or feeling of exhaustion. It reduces quality of life and may negatively affect all occupational and social activities. It may interfere with rehabilitation measures. It can be distinguished from depression, lack of self-esteem, despair, and feelings of hopelessness [6,7], and it is also distinct from limb weakness. Although the pathogenesis is unknown, some investigators have raised the possibility of its relation to the dopaminergic system [5,7–9].

To evaluate the relationship between fatigue and the dopaminergic system, we investigated the effect of pergolide mesilate (a D2 and D1 dopamine receptor agonist) and bromocriptine (a D2 selective dopamine receptor agonist) in patients with PD.

## 2. Methods

41 patients with PD (22 men, 19 women, mean age ± standard deviation [SD] 63.3 ± 10.6 years; range: 50 to 81 years, Table) and 30 age- and sex- matched controls (22 men, 19 women, mean age 62.8 ± 10.0 years), who had been diagnosed in our clinic to have tension headache or migraine without neurological deficits, participated in this study. The control subjects were not demented and did not have cancer, major cardiac diseases, or any other disorders causing major disabilities. Parkinsonian symptoms had been present in patients with PD for 6.1 ± 1.8 years (range: 3 to 10 years) and the disease severity in the "off" state averaged 3.1 ± 0.3 (by the Hoehn and Yahr scale, 30 stage III, eleven stage IV). Patients had been treated with levodopa/carbidopa for 5.7 ± 1.5 years (range: 2 to 10 years). All patients

ISSN 0953-4180/01/02/$8.00 © 2001/2002 – IOS Press. All rights reserved

Table 1
Fatigue severity questionnaire

| | |
|---|---|
| 1. | I feel drowsy when I fatigued. |
| 2. | When I am fatigued, I lose my fatigued. |
| 3. | My motivation is lower when I am fatigue. |
| 4. | When I am fatigued, I have difficulty concentrating. |
| 5. | Exercise brings on my fatigue. |
| 6. | Heat brings on my fatigue. |
| 7. | Long periods of inactivity bring on my fatigue. |
| 8. | Stress brings on my fatigue. |
| 9. | Depression brings on my fatigue. |
| 10. | Work brings on my fatigue. |
| 11. | My fatigue is worse in the afternoon. |
| 12. | My fatigue is worse in the morning. |
| 13. | Performance of routine daily activities increases my fatigue. |
| 14. | Resting lessens my fatigue. |
| 15. | Sleeping lessens my fatigue. |
| 16. | Cool temperatures lessen my fatigue. |
| 17. | Positve experiences lessen my fatigue. |
| 18. | I am easily fatigue. |
| 19. | Fatigue interferes with my physical functioning. |
| 20. | Fatigue causes frequent problems for me. |
| 21. | My fatigue prevents sustained physical functioning. |
| 22. | Fatigue interferes with carrying out certain duties and responsibilities. |
| 23. | Fatigue predated other symptoms of PD. |
| 24. | Fatigue is among my most disabling symptoms. |
| 25. | Fatigue is among my three most disabling symptoms. |
| 26. | Fatigue interferes with my work, family, or social life. |
| 27. | Fatigue makes other symptom. |
| 28. | Fatigue that I now experience is different in quality or severity than the fatigue I experienced before I developed PD. |
| 29. | I experience prolonged fatigue after exercise. |
| 30. | During the past week, I have slept very well. |

satisfied the criteria for PD by Calne et al. [1] and were examined at our neurological clinic between 1998 and 2000. Throughout the study patients maintained their levodopa/carbidopa schedule and took no antidepressant or anticholinergic drugs. All patients did not use benzodiazapine and did not have sleep disturbances [10].

After neurological and neuropsychological examinations, we assessed the degree of fatigue by asking those patients and controls to fill the fatigue scale questionnaire consisting of 30 items [5–7,10]. Each item was a statement about fatigue being rated from 1 to 7. Patients and controls received brief instructions about the questionnaire and filled them out by themselves. Depression was evaluated by the self-assessed depression scale (SDS) of Zung [12] for all patients and controls. The severity of PD was evaluated by the Hoehn and Yahr Scale [2] and the unified Parkinson's disease rating scale (UPDRS) [13]. Cognitive function was screened by the Mini Mental State Examination (MMSE) [14]. In addition to their previously prescribed levodopa/carbidopa, 25 patients received pergolide mesilate (up to 1.75 mg, 990 $\pm$ 357 $\mu$g) and 16 patients received bromocriptine (up to 15 mg, 7.9 $\pm$ 2.5 mg). We increased pergolide mesilate 250 $\mu$g for every four days and bromocriptine 2.5 mg for every four days. We determined the final dose of each drug to observe patients' clinical improvement and side effects if any. There is at least a week before they were assessed. After five weeks, the same examinations were performed. All subjects gave informed consent for the protocol approved by ethical assessment for human study committee in our institution.

All patients were examined by cerebral magnetic resonance imaging(MRIs) to exclude cerebral abnormalities that might affect the clinical manifestations. Each patient gave informed consent for MRI.

For statistical analysis, the Wilcoxon two group signed rank test was used to assess the differences between patients and controls. For correlation analysis, Spearman's rank correlation coefficient was used. All statistical analyses were carried out with a statistic software (STAT View 4.5-J, Hulinks Inc., Tokyo) using a microcomputer (Power Macintosh G3, Apple computer Inc., Cupertino, CA). Statistical significance was defined as $p < 0.05$.

## 3. Results

On brain magnetic resonance imagings (MRIs), no patient showed an abnormal intensity lesion that may affect evaluation. Patients with PD showed normal cognitive function as assessed by the MMSE (mean 27.6 $\pm$ 1.8, range 25–30), but showed significantly higher scores on the fatigue scale score (4.9 $\pm$ 0.7 vs 2.8 $\pm$ 0.8, $p < 0.01$) and the SDS (49.7 $\pm$ 9.9 vs 40.1 $\pm$ 8.0, $p < 0.05$) in comparison to controls.

There was no significant difference between two patient groups (pergolide mesilate vs bromocriptine) concerning age (65.6 $\pm$ 11.0 vs 60.8 $\pm$ 9.2), the Hoehn-Yahr scale (3.24 $\pm$ 0.4 vs 3.04 $\pm$ 0.5), the UPDRS motor scores (29.6 $\pm$ 5.5 vs 29.2 $\pm$ 3.3) and disease duration (6.0 $\pm$ 1.6 vs 6.3 $\pm$ 2.0). Before addition of pergolide mesilate or bromocriptine, there was no significant difference between two patient groups concerning the fatigue scale score (5.1 $\pm$ 0.7 vs 4.8 $\pm$ 0.9) and the SDS (51.8 $\pm$ 11.6 vs 49.5 $\pm$ 4.6). After taking pergolide mesilate or bromocriptine, patients taking pergolide mesilate showed significant improvement in the fatigue scale score (from 5.1 $\pm$ 0.7 to 4.4 $\pm$ 0.55, $p < 0.05$, Fig. 1 ) in comparison to patients taking bromocriptine (from 4.8 $\pm$ 0.9 to 4.7 $\pm$ 0.8, NS, Fig. 1), while neither patient group showed significant

Table 2
Patient characteristic

| | Pergolide mesilate | Bromocriptine | |
|---|---|---|---|
| Number | 25 | 16 | |
| Mean age ± SD year | 65.6 ± 11.0 | 60.8 ± 9.2 | NS |
| Mean PD duration ± SD year | 6.0 ± 1.6 | 6.3 ± 2.0 | NS |
| Mean dopa dose, mg | 184 ± 112 | 194 ± 94.0 | NS |
| Mean pergolide mesilate dose, $\mu$g | 808 ± 388 | 0 | |
| Mean bromocriptine dose, mg | 0 | 12.0 ± 4.2 | |
| Hoehn Yahr scale | 3.24 ± 0.4 | 3.04 ± 0.5 | Ns |
| UPDRS motor score | 29.6 ± 5.5 | 29.2 ± 3.3 | Ns |
| SDS score | 51.8 ± 11.6 | 49.5 ± 4.6 | NS |
| Fatigue scale | 5.1 ± 1.0 | 4.8 ± 0.9 | NS |

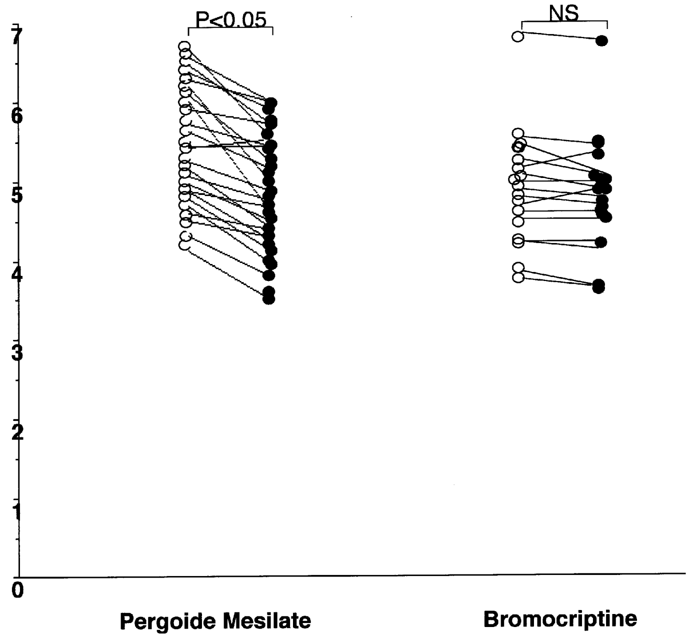

Fig. 1. Fatigue scale scores comparing those at baseline (○) and after taking pergolide mesilate or bromocriptine (•). After taking pergolide mesilate or bromocriptine, patients taking pergolide mesilate showed significant improvement in the fatigue scale scores (from 5.1 ± 0.7 to 4.4 ± 0.55, $p < 0.05$, left) in comparison to patients taking bromocriptine (from 4.8 ± 0.9 to 4.7 ± 0.8, NS, right).

improvement in the SDS (from 51.8 ± 11.6 to 49.4 ± 9.8 and form 49.5 ± 4.6 to 45.5 ± 5.6 respectively, NS, Fig. 2). Both patients groups showed significant improvement in the UPDRS motor scores (from 29.6 ± 5.5 to 26.4 ± 6.6 and from 29.2 ± 3.3 to 23.1 ± 3.0 respectively, $p < 0.05$, Fig. 3). There was no correlation between the severity of depression and the fatigue scale in patients with PD.

## 4. Discussion

In 1993, Friedman and Friedman found that one third of patients with PD rated fatigue as their most disabling symptom and that more than half reported fatigue as one of their three most disabling symptoms. Although fatigue correlated with depression, many nondepressed patients also complained of significant fatigue. They found no correlation between fatigue and disease severity [6]. Using questionnaire, we also found no correlation between the severity of depression and the fatigue scale in patients with PD, although the SDS scores were higher than those in controls [5]. Van Hilten found that 39 (43%) of 90 nondepressed patients with PD reported excessive fatigue with 14 (15%) stating that fatigue was their worst symptom. They found no association between fatigue and disease severity [15]. These results suggested the presence of factors other than depression or disease severity which affect fatigue in patients with PD. Fatigue is often misinterpreted as physical fatigue.

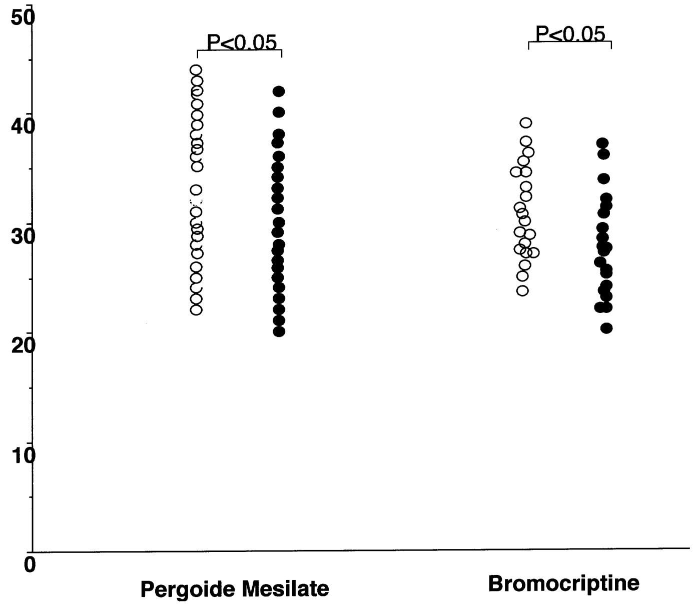

Fig. 2. Zung's Self Rating Depression Scale (SDS) score comparing those at baseline (○) and taking pergolide mesilate (left) or bromocriptine (•) (right). Neither patients group showed significant improvement in the SDS (from 51.8 ± 11.6 to 49.4 ± 9.8 and form 49.5 ± 4.6 to 45.5 ± 5.6, respectively, NS).

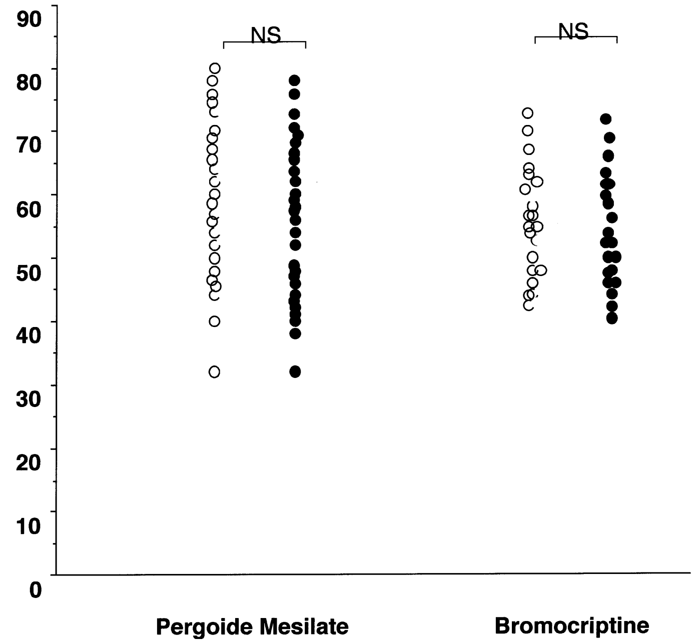

Fig. 3. Unified Parkinson's Disease Rating Scale UPDRS motor score comparing scores comparing those at baseline (○) and after taking pergolide mesilate (left) or bromocriptine (•) (right). After taking pergolide mesilate or bromocriptine, patients taking pergolide mesilate showed significant improvement in the UPDRS motor scores (from 29.6 ± 5.5 to 26.4 ± 6.6 and from 29.2 ± 3.3 to 23.1 ± 3.0, respectively, $p < 0.05$).

However, we as well as previous investigators observed no significant correlation between fatigue and disease severity. In this paper, we defined fatigue as a sense of physical tiredness and lack of energy, distinct from sadness, sleepiness, or impaired motor function secondary to PD [15] and did not distinguished physical fatigue from mental fatigue [16,17].

Fatigue has been correlated with some neurotransmitters. Cohen et al. reported amantadine, which releases intrinsic dopamine and inhibits reuptake, was effective for fatigue in patients with multiple sclerosis [18]. Bruno et al reported bromocriptine might be effective for post-polio fatigue [9]. Recently, attention has been drawn to cognitive dysfunction in PD and dopamine receptors have been associated with cognitive function [19], particularly those in the frontal lobe. Although there is limited knowledge about the type of dopamine receptors having more effect on cognitive function patients with fatigue have shown reduced glucose metabolism or reduced isotope uptake in the frontal lobe, where dopamine receptors are amply present. Thus, it is possibly that improvement of frontal lobe function may improve fatigue [5]. There was a limited number of patients studied, whereas in fact the major drawbacks of the study are the dose equivalence of the two drugs used, the uncertainty in the pharmacological profile of two agents and the fact that the study design was not cross-over. However, our investigation showed the possibility that pergolide mesilate may be effective in improving fatigue.

## 5. Conclusions

Our study suggested the possibility of functional correlation between fatigue and D1 receptor in patients with PD.
